# Gut Microbiota: The Next-Gen Frontier in Preventive and Therapeutic Medicine?

**DOI:** 10.3389/fmed.2014.00015

**Published:** 2014-06-23

**Authors:** Ravinder Nagpal, Hariom Yadav, Francesco Marotta

**Affiliations:** ^1^Division of Laboratories for Probiotic Research, Juntendo University Graduate School of Medicine, Tokyo, Japan; ^2^National Institute of Diabetes, Digestive and Kidney Diseases, National Institute of Health, Bethesda, MD, USA; ^3^ReGenera Research Group for Aging Intervention, Milan, Italy

**Keywords:** dysbiosis, gut microbiota, high-fat diet, metabolic syndrome, microbiome, probiotics

## Abstract

Our gut harbors an extremely diverse collection of trillions of microbes that, besides degrading the complex dietary constituents, execute numerous activities vital for our metabolic and immune health. Although the importance of gut microbiota in maintaining digestive health has long been believed, its close correlation with numerous chronic ailments has recently been noticed, thanks to the innovative mechanistic studies on the compositional and functional aspects of gut microbial communities using germ-free or humanized animal models. Since a myriad of mysteries about the precise structures and functions of gut microbial communities in specific health situations still remains to be explicated, the emerging field of gut microbiota remains a foremost objective of research for microbiologists, immunologists, computational biologists, clinicians, food and nutrition experts, etc. Nevertheless, it is only after a comprehensive understanding of the structure, density, and function of the gut microbiota that the new therapeutic targets could be captured and utilized for a healthier gut as well as improved overall well-being.

Inception: “I then most always saw, with great wonder, that in the said matter, there were many very little living animalcules, very prettily a-moving”.

–Antonie van Leeuwenhoek

Since 1676 when Antonie van Leeuwenhoek first observed the dental microbiota by using his microscope, scientists from all across the globe have always been trying vigorously to explore the diversity and functionality of human-related microbiota. It is now scientifically well-acknowledged that microbes living indigenously in or on the human body perform countless vital functions related to nutrition, metabolism, immunity, diseases, aging, etc., and most of our crucial and fundamental life functions are extensively dependent on our microbiome (1–5). Although extensive literature is available now on the diversity of microbiota associated with our body system, insights into their specific implication and contribution in various physiological functions of human body are just in the beginning stages and there remains a wide array of important issues to be explored and resolved in order to completely understand the complexity of human–microbe relationships. In particular, the role of gut microbiota in health and diseases has been one of the most vigorous and intriguing field of recent researches, although many ambiguities still remain to be elucidated.

Our gut harbors an immensely diverse collection of microorganisms comprising about tens of trillions of microbes, comprising of more than 1000 diverse species of identified bacteria with over three million genes (about 150 times more than human genes). In addition to degrading indigestible dietary components, the gut microbiota is also believed to possess numerous other metabolic abilities and activities that are yet to be discovered or interpreted. Although the gnotobiotic studies have evidenced the significance of gut microbial communities in maintaining normal health and well-being in our life, recent investigations on compositional alterations in gut microbial communities, particularly diet-induced, have revealed an unexpected aspect of the alleged role of gut microbiota in the epidemics of chronic illnesses such as obesity, insulin resistance, type 2 diabetes, metabolic syndrome, inflammatory bowel disease, non-alcoholic fatty liver diseases, atopic allergic disorders, etc. (Figure 1) (5–10). Opportunely, the recent technological advancements in gene sequencing techniques coupled with promising bioinformatics’ tools and omics-based approaches are revolutionizing and aiding in boosting up the extensive and vertical researches on various structural as well as functional aspects of the core human gut microbiome, thereby facilitating the exploration of diverse gut microbial communities and capturing the fundamental changes associated with gut-related ailments. Nevertheless, it is only after an exhaustive comprehension of the diversities, complexities, and functionalities of the gut microbiota that the novel therapeutic targets could be discovered and exploited for better gut as well as overall health (Figure 1).

**Figure 1:**
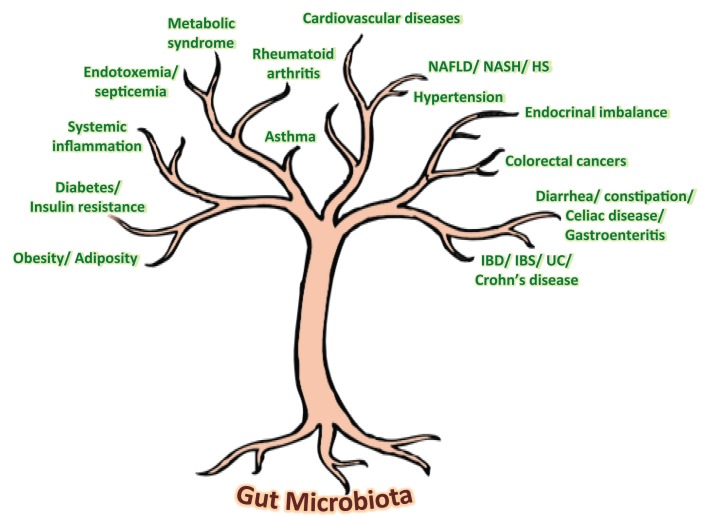
**Speculated health implications of gut microbiota**. NAFLD, non-alcoholic fatty liver disease; NASH, non-alcoholic steatohepatitis; HS, hepatic steatosis; IBD, inflammatory bowel disease; IBS, irritable bowel syndrome; UC, ulcerative colitis.

Since gut microbiota keeps on evolving throughout our entire life, varying from birth to old age, from individual to individual, from healthy to diseased, from children to adults to elderly, etc. (1, 9, 11–14), it is far from settled that what makes a “healthy” or “ideal” gut microbiota profile, particularly when we do not have a model or blueprint of an ideal baseline gut microbiota. While about one-third of our gut microbiota is common to most other humans, the remaining two-thirds (particularly the species composition) may be specific to the individual and may vary in response to our environmental, dietary, and lifestyle influences (7, 9, 11–15). Although ample evidences have emerged out to support the notion that gut microbiota plays an epicentral role in the triangle of diet, health, and diseases, there still subsist a multitude of hidden facts and doubts that remain to be explored and investigated (Figure 2). Obviously, the next step to these predicaments shall be to define a “healthy” infant, adult, and elderly gut microbiota. Moreover, keeping in mind the concept of healthy gut microbiota, it needs to be elucidated what composition of diet and types of dietary macronutrients (for instance, ratios and types of carbohydrates, fats, proteins, etc.) shall be “ideal” to sustain this “healthy” gut microbiota in healthy as well as in a diseased situation? (Figure 2). It does not seem to be easy, since the differences in gut microbiota of healthy and diseased hosts are thin but very complex (9, 11–13, 15). How the diet or dietary components influence the gut microbiota and then how the gut microbiota responds and influences the host metabolism shall be a hectic task to elucidate. Undoubtedly, plentiful data are gradually accumulating to indicate that the gut microbiota could significantly influence a number of metabolic pathways in the host nutrition, including carbohydrate, lipids, bile acids, short-chain fatty acids, vitamins, energy extraction, fat storage, etc. (3–6, 10, 13, 15). It can easily be summed up from these established and acknowledged facts that we share a delicate and straight give-and-take relationship with our gut microbiota (quote: “*we give it good, it gives us good; we give it bad, it may give us worse*”). Furthermore, although numerous reports are available to indicate the therapeutic role of dietary supplements on gut microbiota-mediated health effects, more trials are anticipated to illustrate and authenticate the mechanistic and clinical influences of these supplements such as probiotics, prebiotics, antibiotics, herbal compounds, or nutraceutical interventions on gut microbiome and overall health (16–18). Another hurdle could be to identify and separate out the beneficial, commensal, and harmful microbes out of the extremely complex gut microbial world. More particularly, identifying the beneficial, harmless, and harmful microbes, delineating the effects of surplus beneficial bacteria and too less pathogenic bacteria, and then puzzling out how much of whom is “too much” or “too less” seems to be a tricky and laborious task, especially in view of the fact that each and every member of the gut microbial ecosystem is significant to the overall composition of gut microbiota makeup (14, 19, 20). Obviously, it is only after we know the precise role of each and every member of the gut microbial family that we could manipulate it by reducing the harmful ones either with the beneficial ones or targeted therapeutics or both in order to upgrade the concept of gut microbiota from disease predisposition to disease prevention and treatment without affecting the metabolic and physiological health.

**Figure 2:**
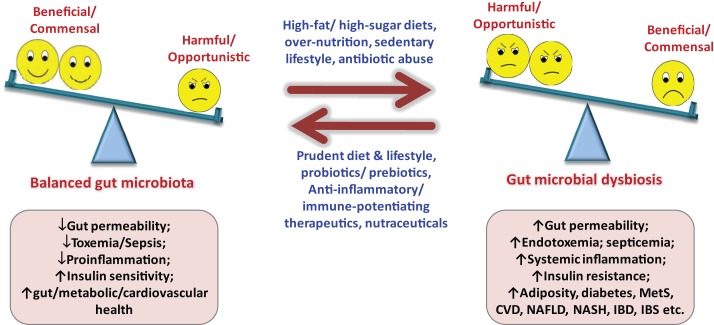
**Importance of balanced nutrition and gut microbiota, and consequences of gut dysbiosis**. MetS, metabolic syndrome; NAFLD, non-alcoholic fatty liver disease; NASH, non-alcoholic steatohepatitis; IBD, inflammatory bowel disease; IBS, irritable bowel syndrome; CVD, cardiovascular diseases.

Although it is evident now that gut microbial alterations are linked with several ailments (5–7, 10, 12, 13), it is still not completely explained whether gut microbial alterations produce the disease or it is the other way around. Again, it is only after sketching out the healthy gut microbial makeup that we could imagine prospective nutrition-based therapies intended to normalize the altered gut microbiota to a normal one (Figure 2). Another important link between gut microbiota and diseases is gut permeability, and it shall be prerequisite to explicate whether altered gut microbiota is causing the disease or the injured gut permeability or both, particularly in maladies associated with “leaky gut” such as insulin resistance, type 2 diabetes, inflammatory bowel diseases, irritable bowel syndrome, celiac diseases, etc. (Figure 2) (7). The new concept of fecal transplant is also in unsteady status, and is going to be tested further in animal as well as clinical settings. Another common question which needs to be addressed comprehensively is whether 1 g of fecal sample could truly represent the gut microbiota, since the flora of lower intestinal sample may differ (slightly or largely) from that of actual intestinal environment. In addition, there is a variation in the microbial diversity in distal colon to that in upper gut or other parts of the large intestine. Moreover, there might be some variation in the bacterial relative abundance within one sample but at different sampling spots. Also, using the gut microbiota signature for diagnosis and targeting it for prevention as well as treatment of an ailment such as obesity, insulin resistance, metabolic syndrome, type 2 diabetes, hepatic steatosis, bowel diseases, etc., needs to be explicated further. The relationship between infant’s and mother’s gut microbiota still remains ambiguous, and needs to be verified in healthy as well as abnormal settings, in addition to the effect of mother’s vaginal microflora and milk components such as fatty acids and hormones on infants gut microbial health. Also, how the gut microbiota at-birth relates to the health and disease predisposition during later years seems to be intriguing (9). Nonetheless, with the advent of advanced next-gen sequencing tools and technologies and rigorous progresses in mechanistic studies of the human microbiome project via gut microbiome, metagenome, metatranscriptome, metaproteome, metabolome, inflammasome, etc., these doubts and challenges are appearing to be deciphered more quickly than expected in near future (14, 19–21).

Though, the research on human gut microbiota is succeeding logarithmically, the field still remains an emerging and in-progress area of research. Our gut microbiome includes more than three million genes, however, we do not know much about the functions or interactions of most of these genes (20). Investigators are making incredible advances in comprehending not just what the gut microbiota does or can do, but also how these microbes do whatever they do, how this mystifying group of gut microbes could positively and negatively impact our nutrition, physiology, healthiness, and diseases, and how can we can manipulate or engineer it for better clinical health. Certainly, it can easily be envisaged that this evolution of research on gut microbiota shall revolutionize the notion of “we are what we eat” to “we are what our gut microbiome is.” Optimistically, we may soon witness an era where the gut microbiome clinics shall be prevalent all around and the individual’s gut microflora will be widely used as a diagnostic, prophylactic as well as therapeutic target for a myriad of health problems, and more fascinatingly, the gut microbiome at infant stage shall be used to predict predisposition to numerous ailments in later years, and individuals’ diet regimens shall be designed exclusively according to their gut microbiota profile for a better and disease-free health and well-being. In the meantime, in all this evolving setting, the practicing clinicians, so far excluded by any objective understanding of gut milieu changes, are receiving ample help from a number of reliable and precise non-invasive diagnostic tools to scrutinize gut microbiota, dysbiosis, gut inflammation, and gut permeability which is proving to be valuable in clinical practice to unveil subtle disease risk-prone conditions, shape up a tentative-tailored intervention, and maintain an objective follow-up monitoring.

## Conflict of Interest Statement

The authors declare that the research was conducted in the absence of any commercial or financial relationships that could be construed as a potential conflict of interest.
